# Physical activity patterns among women during the postpartum period: an insight into the potential impact of perceived fatigue

**DOI:** 10.1186/s12884-022-05015-0

**Published:** 2022-09-03

**Authors:** Baian A. Baattaiah, Haya S. Zedan, Arwa S Almasaudi, Shoug Alashmali, Monira I. Aldhahi

**Affiliations:** 1 Department of Physical Therapy, Faculty of Medical Rehabilitation Sciences, King Abdulaziz University, Jeddah, Saudi Arabia; 2 Department of Public Health, College of Health Sciences, Saudi Electronic University, Riyadh, Saudi Arabia; 3 Department of Clinical Nutrition, Faculty of Applied Medical Sciences, King Abdulaziz University, Jeddah, Saudi Arabia; 4 Department of Rehabilitation Sciences, College of Health and Rehabilitation Sciences, Princess Nourah bint Abdulrahman University, P.O. Box 84428, Riyadh, 11671 Saudi Arabia

**Keywords:** Physical activity, Fatigue, Postpartum, Maternal health

## Abstract

**Background:**

Regular participation in physical activity (PA) improves physical well-being and reduces the risk of contracting noncommunicable diseases. However, fatigue could negatively impact the PA participation of women in their postpartum period. This study delineated the levels of perceived fatigue and characterized the association between fatigue and the PA patterns of postpartum women.

**Methods:**

A cross-sectional study was conducted using an online questionnaire distributed to postpartum women living in Saudi Arabia. Their perceived postpartum fatigue (PPF) was assessed using the fatigue severity scale; their PA, using the short form of the International Physical Activity Questionnaires; and their postpartum depression, using the Edinburgh Postnatal Depression Scale. Descriptive statistics were expressed as the mean ± standard deviation for normally distributed variables and as the median (interquartile range) for non-normally distributed variables. Between-group differences were tested using the Mann–Whitney U test for independent samples. To determine the relationship between the study variables, Spearman’s rho correlation coefficient was calculated. Multiple linear regression analysis was performed to explain the role of fatigue severity as an independent predictor of the variance of the PA level.

**Results:**

A total of 499 postpartum women were divided into the PPF group (43%), who self-reported fatigue, and the non-PPF group (57%), who self-reported no fatigue. There was a significant difference in the median of vigorous PA, and moderate PA which were significantly higher in the non-PPF group than in the PPF group. The women with PPF reported less engagement in walking and a longer sitting duration than the women without PPF. High fatigue severity was associated with lower moderate PA (β = -10.90; *p* = .005; *R*^*2*^ = .21) and vigorous PA (β = -04; *p* < .001; *R*^*2*^ = .13). These associations remained significant in the regression model after adjustment for the mother’s depression score; age; number of children; body mass index (kg/m^2^); employment status; intake of vitamins B1 (thiamin), C, and D and of Omega-3; and walking metabolic equivalent.

**Conclusion:**

PPF may reduce the PA of postpartum women. Strategies targeting PPF may buffer its harmful impacts, and thus, improve postpartum women’s health.

## Background

The postpartum period (PPP) is considered a transitional time during which women and their families need to adjust the physical, psychological, and social aspects of their lives [1]. The PPP has also been defined as the “fourth stage of labor” that has three distinct but continuous phases: the acute, subacute, and delayed phases, from immediately post-birth and up to 6 months after [2]. During these phases, the body undergoes major physiological, emotional, and functional changes. Some changes take a lot longer to resolve, and some might never fully return to their pre-pregnancy status [2, 3]. Therefore, multiple guidelines, including from the World Health Organization (WHO), stress the significance of early postpartum follow-up with an experienced clinician, particularly after departure from the hospital following childbirth, to minimize postpartum morbidity [4–6]. It is recommended that women utilize specialized postpartum support strategies and services to improve their maternal knowledge, attitudes, and abilities pertaining to parenting, maternal mental health, quality of life, and physical health [7–9] .

Recommendations for regular physical activity (PA) during the PPP have been well documented in literature [10–12]. PA is generally defined as any bodily movement caused by the contraction of skeletal muscles [13]. Regular PA in *all* phases of life improves physical well-being, lowers the risk of contracting noncommunicable diseases (NCDs; e.g., cardiovascular disease [14], diabetes mellitus [15], obesity [16], cancer [17], lipid profile abnormality[18], and bone density disorders [19]), and lowers the total mortality rate [20]. Furthermore, research has found that PA is linked to better mental health [21], pleasant mood [22], fewer depressive symptoms [23], and a positive affective state on general well-being [24]. In contrast, physical inactivity contributes to chronic stress and poor mental health [25–28]. The Centers for Disease Control and Prevention has declared that the abovementioned benefits also apply to pregnant and postpartum women [12]. Recent WHO recommendations affirm the United States Department of Health and Human Services Physical Activity Guidelines of at least 150 minutes of moderate-intensity aerobic activity per week throughout pregnancy and the PPP [5]. For women who were physically active prior to pregnancy, the safety and suitability of maintaining these activities during pregnancy and the PPP depend on their health status and the supervision of their healthcare provider [29]. Despite the known widespread benefits of PA, several studies have reported women’s lack of participation in PA after childbirth [30, 31].

A systematic review of PA behaviors revealed a high prevalence of inactivity, particularly among women [32]. Bauman et al. [33] conducted a study to compare the international prevalence of PA across 20 countries; the results also confirmed the low prevalence of PA among women when compared to men. Another study that measured daily step counts revealed a significant level of inactivity among the female participants based on the international norm for minimum activity [34]. In the US [35] and Australia [36], it has been found that a fair percentage of women do not engage in regular PA. The tendency of women to not engage in PA tends to be consistent across the different phases of life but lower during the PPP [30, 31]. This may be because postpartum women seem to devote most of their time to providing adequate care to their children, adopting appropriate parenting behaviors, and developing strong bonds with their infants [37–39]. These activities may reduce the time available for PA in this period and thus, escalate women’s risk of developing NCDs and other devastating disorders such as depression and anxiety [27].

Along with time constraints, it is clear that taking care of a newborn baby necessitates both physical and mental effort. Early in life, babies need to be fed, need consistent monitoring and many exabit irregular sleep patterns [40, 41]. In addition, mothers experience drops in hormone levels after childbirth, which cause physiological changes during the PPP [42]. Collectively, these factors may contribute to the development of postpartum fatigue (PPF) [43, 44]. PPF has been associated with negative consequences for the mother-infant relationship [45], daily life activities [46], and overall maternal well-being and quality of life [47]. Despite debate over the precise definition of fatigue, it is best described as a state marked by feelings of tiredness or weariness and a decrease in physical or mental performance [48, 49]. Although *fatigue* and *tiredness* are often used interchangeably, fatigue is considered a more severe and negative feeling that lasts longer and is not easily relieved [50, 51]. On the other hand, PPF is a multidimensional concept with physical, emotional, and cognitive components [50]. A mother can experience physical fatigue as exhaustion and lethargy; emotional fatigue, as anxiety or depression; and cognitive fatigue, as lack of interest and focus [50, 52].

Research on maternal health has mainly focused on PPF [43–45, 50], postpartum depression (PPD) [53, 54], and the effect of exercise on these outcomes [55, 56]. Little is known, however, about the predicted effect of fatigue on PA patterns during the PPP, which should be examined to prevent health-related complications. Therefore, this study delineates the severity of maternal perceived fatigue and characterizes the association of fatigue with PA patterns in mothers during the PPP. The findings contribute to the fundamental knowledge about women’s health and, specifically, the effect of fatigue on PA during the PPP. From a clinical perspective, the findings could be used to develop and implement strategies e.g., rehabilitative, which target fatigue to maintain and improve the health and PA patterns of this group of women during this critical period.

## Methods

### Study design

A cross-sectional study was conducted in all Regions of Saudi Arabia between February 2021 and August 2021. It was conducted according to the guidelines proposed in the Declaration of Helsinki and was reviewed and approved by the Unit of Biomedical Ethics Research Committee, Faculty of Medicine, King Abdulaziz University, Jeddah, Saudi Arabia (Registration Number: HA-02-J-008, Reference No 84-21).

### Sample size calculation

A priori sample size was calculated using the OpenEpi.com. Based on the total Saudi female population in 2020 [57] and with the confidence level set at 95%, the margin of error at 5%, and the anticipated frequency at 50%, the estimated sample size required for this study was 385 participants.

### Study participants and recruitment

**Fig. 1:**
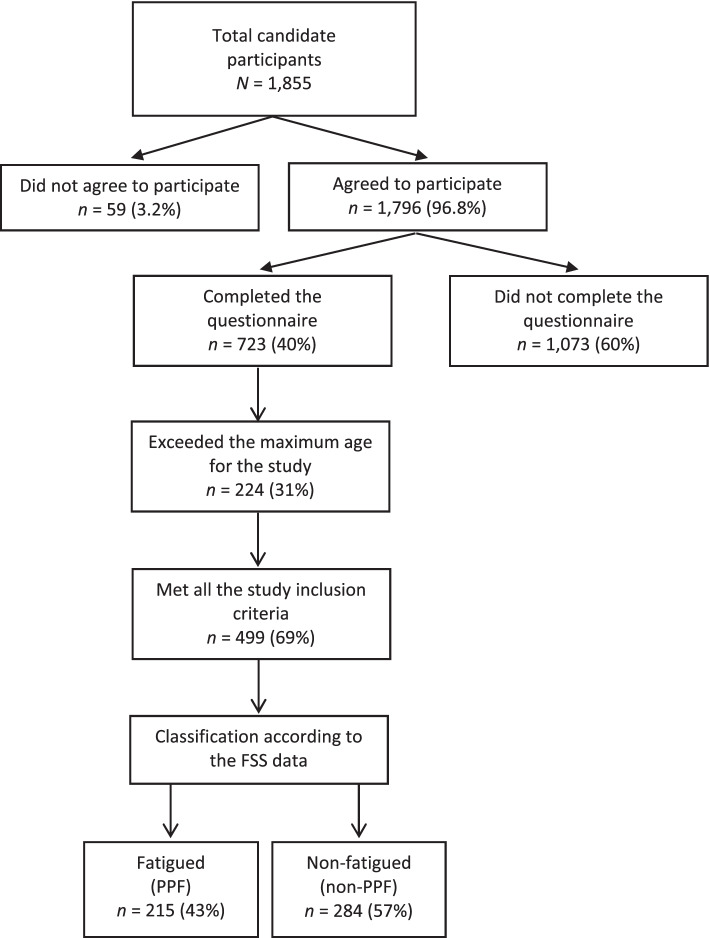
Flow diagram of the study recruitment

A total of 499 postpartum women were included in the final analyses. They were all between 18 and 50 years old, living in Saudi Arabia, within the first year after childbirth, and had no history of mental illness, cardiovascular disease, and physical disability. An electronic questionnaire was created using an online survey platform (SurveyMonkey®). The link to the survey was distributed *via* email and through various social media platforms (WhatsApp, Twitter). A reminder to participate in the study was sent on a regular basis to improve the study recruitment. Participants were allowed to answer the questionnaire only once. The study protocol, procedures, and participants’ rights were explained at the beginning of the survey and written informed consent was obtained from all participants prior to their participation. Of the 1855 who responded to the survey, 59 (3.2%) did not agree to participate in the study, 1073 (60%) did not complete the entire questionnaire, and 224 (31%) were excluded because they exceeded the maximum age for study participation. According to the fatigue severity scale (FSS), participants were classified into fatigued (PPF) and non-fatigued (non-PPF) groups. The flowchart in Fig. 1 shows the process of recruitment and classification of the study participants.

### Data collection

All the participants completed the self-administrated electronic questionnaire that included the following: questions on their demographics, employment status, family-related factors, pregnancy-related factors (intake of vitamins and minerals, age, number of children, mode of childbirth, age of the baby, and breastfeeding mode), health-related factors (mother’s PPD score, smoking status, medical condition, and body mass index [BMI]), and the fatigue severity scale (FSS), and the short form of the International Physical Activity Questionnaire (IPAQ-SF).

### Fatigue severity scale (FSS)

The participants’ fatigue was assessed using the FSS. This questionnaire was first developed by Krupp et al. [58] and includes nine items that assess the effect of fatigue on daily living [58, 59]. Each item is a statement on fatigue that the subject rates from 1 (*completely disagree*) to 7 (*completely agree*). The questionnaire is brief, easy to administer, and demonstrates reliability and internal consistency. Participants with a mean score of 4 or more were defined as experiencing significant fatigue. The translation of the FSS into Arabic was performed according to international standards, and the resulting tool is valid for use by Arabic-speaking populations [60].

### Physical activity (PA) questionnaire

The IPAQ-SF [61] was used in this study. It is a self-reporting 7-day recall of physical activity designed to provide information about duration and frequency of engagement in different pattern of physical activity in the last seven days. The IPAQ-SF categorizes physical activities into four generic groups: vigorous, moderate, walking, and sitting. The participants are rated according to the following three PA categories: Active (vigorous-intensity activities for at least three days per week of a totaling at least 1,500 MET-min/week; or seven or more days of any combination of walking, moderate-intensity, or vigorous-intensity activities equivalent at least 3,000 MET-min/week); minimally active (indicated engagement in vigorous activity of at least 20 min per day for three or more days or engaged in five or more days of moderate-intensity activities or walking for at least 30 min per day, or five or more days of any combination of walking, moderate-intensity, or vigorous-intensity activities totaling at least 600 MET-min/week); inactive to insufficiently active (defined as not meeting criteria of minimal active or high active).

The use of the IPAQ-SF allows for the calculation of metabolic equivalents (METs), which are the energy requirements for each type of activity. Following existing guidelines, the IPAQ-related variables are reported in minutes per week (min/week) and in METs (METs [min/week]) [62]. The concurrent validity of the IPAQ-SF shows reasonable agreement with the long-form questionnaire, and its criteria validity reveals a fair-to-moderate agreement with the Computer Science Application, Inc. accelerometer [63, 64].

### Edinburgh Postnatal Depression Scale (EPDS)

The EPDS has been validated and translated into 18 languages, including Arabic [65]. This questionnaire is used to screen participants’ depression levels for further assessments and referrals for treatment. The EPDS is a 10-item self-reporting scale used to assess the common symptoms of depression. Each item is scored on a 4-point scale (0–3), and the total scores can range from 0 to 30 [66]. The cutoff value of ≥ 12 has been shown to indicate good specificity for PPD in Arabic-speaking cultures [67].

### Data analysis

The collected data were analyzed using the Stata (version 16.0) software package (StataCorp, College Station, Texas, USA). A descriptive statistical analysis of the data was conducted to determine the distributions of the sociodemographic and general health variables. The normality assumption was tested using the Shapiro–Wilk test. Descriptive statistics were expressed as mean ± standard deviation (SD) values for normally distributed variables and as median (MED) and interquartile range (IQR) values for non-normally distributed variables. Between-group differences were tested using the Mann–Whitney U test for independent samples. The Spearman’s rho correlation coefficient was calculated to determine the relationship between the fatigue severity score and the PA level. Multiple linear regression analysis was performed to explain the role of fatigue severity as an independent predictor of the variance of the PA level. The analysis model 1 of regression was controlled for the respondent’s depression score, age, number of children, BMI, employment status, and intake of vitamins B1 (thiamin), C and D and of Omega-3, whereas the full model was controlled for all the variables in model 1 plus the walking METs. The significance level was set at a *p*-value of < .05 for all the statistical analyses in this study.

## Results

### Participant characteristics

Of the 499 postpartum women included in this study, 215 (43%) reported high self-perceived fatigue and 284 (57%) did not perceive any fatigue during activities. The women were divided into two groups: the fatigued group (PPF) and the non-fatigued group (non-PPF). The sociodemographic characteristics and pregnancy-related factors of the participants are presented in Table 1. Most of the sociodemographic characteristics of the groups did not significantly differ. However, the groups significantly differed in income, employment status, and educational level. An average income of less than 10,000 riyals was reported in both groups. Most of the participants had a bachelor’s degree and a high job status. The mean age and the depression scores of the women in the PPF group did not significantly differ from those in the non-PPF group (*p* = .28 and *p* = .11, respectively). There were significant differences between the groups in their intakes of vitamins B1 (thiamin), C, and D and of Omega-3.

**Table 1:** Sociodemographic characteristics and pregnancy-related factors of the participants

**Variable**	**PPF** *n* = 215	**Non-PPF** *n* = 284	***p*-value**
**Weight** (kg)	66 (13)	66 (16)	.64
**Height** (cm)	161 (7)	160 (7)	.62
**Body mass index** (kg/m^2^)	26 (4.7)	25.8 (5)	.77
**Baby age** (months)	7 (8)	3 (6)	< .001*
**Mother age** (years)	29 (6)	30 (6)	.28
**Postpartum depression score**	14 (6)	15 (5)	.11
**Income** (Saudi riyal, n, %)			
< 10,000	127 (59.07)	212 (74.7)	.001*
10,000–20,000	58 (26.98)	52 (18.3)	
20,001–30,000	22 (10.23)	11 (3.87)	
> 30,000	8 (3.72)	9 (3.17)	
**Number of children per mother** (n, %)			
1	50 (23.26)	57 (20)	.17
2	93 (43.26)	143 (50.35)	
3	42 (19.53)	59 (20.77)	
4	30 (13.95)	25 (8.80)	
**Educational level** (n, %)			
No degree	5 (2.33)	10 (3.52)	.004*
High school or less	26 (12.09)	19 (6.69)	
Bachelor’s degree	156 (72.56)	221 (77.8)	
Postgraduate degree	28 (13.02)	34 (11.9)	
**Smoker** (n, %)	13 (6.06)	11 (3.87)	.26
**Breastfeeding status**			
Yes	100 (46.51)	148 (52.11)	.24
Sometimes	85 (39.53)	97 (39.11)	
No	30 (13.95)	39 (13.73)	
**Employment status** (n, %)			
Employed	128 (59.53)	201 (70.77)	.009*
Unemployed	87 (40.47)	83 (29.23)	
**Delivery mode** (n, %)			
C-section	58 (26.98)	56 (19.72)	.06
Vaginal	157 (73.02)	228 (80.28)	
**Housemaid assistant** (n, %)			
Yes	161 (74.88)	220 (77.47)	.18
No	54 (25.12)	64 (22.54)	
**Vitamin B1 (thiamin)** (mg/day)	1.3 (2.91)	1.5 (3.9)	.03*
**Vitamin C** (mg/day)	106.7 (177)	97.7 (130)	.0001*
**Vitamin D** (mg/day)	0 (0–5000)	1000 (0–5000)	.0001*
**Omega-3** (mL)	0 (200)	200 (250)	
**Iron** (mg/day)	23.7 (38.7)	27.7 (43.8)	

^*^Significance level of *p* ≤ .05

*Abbreviations*: *PPF,* Postpartum fatigue; *n,* Frequency; *(%),* Percentage

### PA and fatigue characteristics

Table 2 compares the PA levels of the two groups. There was a significant difference in the vigorous PA median (*p* < .001); the women without fatigue reported engaging in vigorous PA twice as much per week as the women who reported a high level of fatigue. The moderate PA level was significantly higher among the women in the non-PPF group than those in the PPF group (*p* < .001). The median fatigue severity score in the PPF group was significantly higher than the cutoff value (≥ 4), which was reported previously to indicate severe fatigue. There was a difference between the total METs of the PA of the groups, with the total METs in the PPF group 260 min/week lower. The women with PPF reported less engagement in walking and a longer sitting duration than the women without PPF.

**Table 2:** Comparison of the physical activity levels and the self-reported fatigue severity of the postpartum fatigue (PPF) and non-PPF groups

**Variable**	**PPF** *n* = 215		**Non-PPF** *n* = 284		***p*-value**
	**Mean**	**MED (IQR)**	**Mean**	**MED (IQR)**	
Vigorous PA (min/week)	25.2	0 (20)	46.8	20 (20)	< .001*
Moderate PA (min/week)	113.9	20 (80)	125.4	45 (100)	< .001*
Walking (min/week)	168.26	60 (210)	186.2	120 (145)	.0003*
Sitting (hr)	8	6 (6)	5	6 (3)	.02*
Total METs (min/week)	1213.5	596 (1425)	1491.7	856.5 (1203.5)	< .001*
FSS	5	5 (1.5)	3	3 (0.7)	< .001*

^*^Significance level of *p* ≤ .05; Mann–Whitney U test was used for statistical analysis

*Abbreviations*: *n,* frequency; *MED,* Median; *IQR,* Interquartile range; *PA,* Physical activity; *METs,* Metabolic equivalents; *FSS,* Fatigue severity score

Most of the women in the PPF group (123 [57%]) had low PA levels, which indicated that they did not meet any of the criteria for moderate and vigorous PA, unlike the women in the non-PPF group (99 [34%]). In contrast, a higher proportion of the non-PPF women indicated that they engaged in moderate PA compared to the PPF women (120 [42%] vs. 57 [27%]).

### Association between fatigue severity and PA patterns

Table 3 shows that fatigue severity was negatively correlated with the PA pattern. The sitting duration was found to have a moderate positive correlation with fatigue severity. Fatigue severity had no significant correlation with the EPDS scores. The multiple linear regression model of the association of vigorous and moderate PA, as dependent variables, with fatigue severity is illustrated in Table 4. Severe fatigue was associated with lower vigorous PA (β = -04; *p* < .001; *R*^*2*^ = .13) and lower moderate PA (β = -10.90; *p* = .005; *R*^*2*^ = .21) after controlling for the mothers’ depression scores; age; number of children; BMIs; employment status; intakes of vitamins B1 (thiamin), C, D and of Omega-3 (mL), and walking time. Thus, there was no evidence of multicollinearity in any of the models.

**Table 3:** Matrix of the Spearman correlation between the fatigue severity score and physical activity levels and depression

**Variable**	**Correlation Coefficient**						
	**1**	**2**	**3**	**4**	**5**	**6**	**7**
FSS	1						
Total PA (min/week)	-0.14**	1					
Vigorous PA METs (min/week)	-0.098*	0.22**	1				
Moderate PA METs (min/week)	-0.12**	0.66**	0.01	1			
Walking METs (min/week)	-0.08 *	0.65**	-0.06	0.45**	1		
Sitting (hr)	0.26**	-0.10*	-0.06	-.009*	-0.06	1	
EPDS	-0.06	-0.07	0.59	0.67	-0.08	-0.07	1

^*^Correlation is significant at *p* < .05. ** Correlation is significant at *p* < .01

*Abbreviations*: *FSS,* Fatigue severity score; *PA,* Physical activity; *METs,* Metabolic equivalents; *EPDS,* Edinburgh Postnatal Depression Scale

**Table 4:** Association of fatigue severity as an independent variable with metabolic equivalents of vigorous- and moderate-intensity physical activity

**Model**		**β**	**SE**	**T**	***p-*value**	**95% CI**		**F**
						**Lower**	**Upper**	
Vigorous PA (min/week)								
FSS	Model 1^a^	-.04	.006	-6.04	< .001	-.05	-.02	35.77
	Model 2^b^	-.04	.006	-6.04	< .001	-.05	-.02	36.44
Moderate PA (min/week)								
FSS	Model 1^a^	-14.72	4.20	-3.51	.001	-22.98	-6.47	12.29
	Model 2^b^	-10.90	3.88	-2.81	.005	-18.53	-3.27	7.89

^a^Analysis adjusted for the depression score (using the Edinburgh Postnatal Depression Scale); age of the mother; number of children per mother; body mass index (kg/m^2^); employment status; and intakes of vitamins B1 (thiamin), C and D (mg/day) and of Omega-3 (mL)

^b^Analysis adjusted for Model 1 plus walking METS

*Abbreviations*: *β,* Standardized beta; *SE,* Standard error; *CI,* Confidence interval; *PA,* Physical activity; *FSS,* Fatigue severity scale

## Discussion

The overarching aims of this study were to describe the PA patterns and perceived fatigue severity and to understand the extent of the association of PA with fatigue during the PPP. The findings of this study highlight the severity of fatigue among postpartum women and its influence on PA and raises concerns about PA patterns among women with PPF. Most women with self-reported fatigue revealed a low level of PA, unlike the women without self-reported fatigue. The postpartum women with fatigue reported higher sitting durations and lower levels of vigorous and moderate PA than the postpartum women without fatigue. The women with fatigue were found to have lower MET values than those without fatigue. These results confirm the association between fatigue severity and PA. Fatigue severity has been shown to explain the variance in moderate and vigorous PA levels among postpartum women. These findings provide a foundation for the promotion by clinicians and PA practitioners of the inclusion of PA interventions for postpartum women to improve their perceived fatigue severity.

During the PPP, women are susceptible to many physiological and mental changes, which lead to shifts in the dynamics of their lifestyles. PPF has been reported to occur in 11.4% of women three months after childbirth. Its degree has been shown to vary according to the woman’s age, education level, and income [45]. Little is known about PA patterns during the PPP, which may be jeopardized by fatigue severity. However, it is known that PA during the PPP is an important factor of psychosocial well-being and physical health [12]. As stated earlier, the WHO PA guidelines recommend that postpartum women engage in at least 150 minutes of moderate-intensity aerobic PA per week [6]. Blum et al. [68] found that postpartum women’s engagement in PA and sporting activities enhances their well-being. In addition, Nakamura et al. [69], confirmed that postpartum PA helps women to overcome negative mood states and symptoms of depression. Despite the positive health outcomes associated with PA engagement, our study found a reduction in the PA METs of women who reported PPF compared to those of women who reported no PPF. Saligheh et al. [70] identified the barriers to PA as environment-related factors and personal factors related to the mother’s own circumstances. The two most common barriers were lack of time and lack of access to appropriate and affordable exercise facilities. Although our results, in terms of PA, agreed with the results of Saligheh et al., fatigue was not considered an impeding factor of PA engagement.

Fatigue is a global concern of postpartum women, and this study found a large percentage of fatigue prevalence among the participants. This finding is in agreement with those of previous studies [71–73] in which fatigue was frequently reported by mothers after childbirth. The results also concur with those of Henderson et al. [45], who conducted a large population-based study and assessed fatigue at three different times: at 10 days, one month, and three months postpartum. They found that fatigue affected a substantial proportion of postpartum women (38.8%, 27.1%, and 11.4% experienced fatigue or severe tiredness 10 days, one month, and three months postpartum, respectively). Our results provide an insight into the influence of fatigue on PA that can be used to direct future provision of appropriate, tailored, and individualized rehabilitative programs for postpartum women. Studies should also be conducted to examine the mechanisms by which fatigue severity may reduce PA.

Based on literature, there are many potential factors that could explain this trend of fatigue among postpartum women. Studies have shown that the mother’s age, number of children, educational level, presence of postpartum comorbidities, breastfeeding mode, and availability of assistance and a support system predict the development of PPF [45]. In addition, the sleep patterns of mothers and infants have been shown to contribute to the development of fatigue and to diminish the daytime functioning of postpartum women [45, 74]. Interestingly, sudden physiological changes related to childbirth have been shown to be potential mediators of an increased risk of PPF [75]. In this study, factors such as the mother’s depression score; age; number of children; BMI; employment status; intake of vitamins B1 (thiamin), C, and D and of Omega-3; and walking time were all controlled, but the relationship between fatigue and PA was still significant. However, investigating the factors contributing to fatigue development among our participants was beyond the scope of this study. Future studies are recommended to explore the mediating factors of PPF in postpartum women.

During the PPP, a mother’s level of fatigue impacts her level of PA. PPF and a general lack of energy have been reported to impede participation in leisure-time PA [39, 76]. Our results revealed a negative relationship between fatigue severity and PA, and the role of PPF as a predictor of vigorous and moderate PA. Regression analyses that focused on the predictors of PA levels revealed that more severe PPF is associated with lower engagement in moderate and vigorous PA. Women who reported higher levels of fatigue demonstrated lower levels of PA than women who reported lower levels of fatigue. The trend of these findings is feasible, as it is similar to results shown in the study of Haas [77] regarding fatigue and PA among women with specific chronic conditions and those of a study by Egerton et al. [78] in older adults population. Although, PPP is different than the aforementioned conditions, yet those studies together reasonably confirm the existence of such a relationship between fatigue and PA.

### Strengths and limitations

To the best of our knowledge, this study was the first to investigate the effect of PPF on the patterns of PA in postpartum women in Saudi Arabia. A large random sample size was used from all regions of Saudi Arabia. The study shed the light on the potential impact of fatigue on PA levels among women during PPP, which may provide insight into rehabilitative strategies to promote maternal health. However, this study had several important limitations. First, the nonprobability convenience sampling method used may influence the generalizability of the findings to a representative sample of women in Saudi Arabia. Second, the PA and fatigue severity measurements relied on self-reporting rather than objective measures. Further study is required to investigate the mechanism underlying PPF using blood-based biomarker of women and to establish cause-and-effect relationships.

Third, the subjective measurement of the PA and fatigue levels might have led to reporting bias due to variations in the women’s perception and assessment of their experiences. As a result, the dissemination of the study findings might have been tainted in some way. Using objective measurement tools (e.g., pedometers, accelerometers, Fitbits, or digital wristwatches) to quantify PA would generate more applicable data and be a more rigorous methodology.

Fourth, all our participants were recruited through electronic means, which might have led to selection bias and hindered the generalizability of our findings. Future studies should consider either direct interviews or objective data collection methods to prevent such problems.

Fifth, the study investigated the influence of fatigue on PA. However, we did not provide enough information related to the other perceived factors, such as socioeconomic and environmental, self-efficacy, resilience, self-esteem factors, which may also have contributed to PA during the PPP. Additionally, we did not investigate the factors that might have contributed to the development of postpartum fatigue among our participants. Further studies should expand on this matter to provide an overarching view of the potential contributing factors of PA and fatigue levels among postpartum women. Sixth, we did not investigate the baseline levels of fatigue during and prior to the women’s pregnancy. Such information is important to compare the PA and fatigue levels before and after childbirth.

An additional limitation is that we did not collect information about the medications that the participants were taking or the risk factors of engaging in PA for this cohort. Both these factors could have indirectly influenced the PA and fatigue levels. Moreover, we did not investigate the women’s PA levels before they gave birth. Such information could have expanded our knowledge of the impact of fatigue on PA levels. Therefore, a study to compare PA levels before, during, and after childbirth is recommended. Finally, further studies should consider effective strategies for the recruitment and motivation of study participants to minimize dropouts and thus, generate a larger representative sample.

## Conclusions

In this study, it was reported that the participants’ engagement in different levels of PA was low during the PPP. Fatigue was found to be a potential contributing factor of this reduction in PA. A high percentage of the participants reported a high level of PPF. We found that PPF is a strong predictor of vigorous and moderate PA and that higher fatigue levels are associated with lower engagement in moderate and vigorous PA. Our findings highlighted the predictive effect of the level of fatigue, which explained 21% of the variance in the women’s moderate-level PA and 13% of the variance in their vigorous-level PA. Our data give insights on which early and appropriate interventions to reduce PPF could boost the mitigation of its negative effects on PA, and therefore, could improve postpartum maternal health. Our findings also suggest that other factors may have an impact on PA and thus, require further investigation.

It is necessary to place greater emphasis on the role that PA plays throughout the PPP. Following the weekly PA recommendations is critical for health improvement, as a progressive fall in PA can threaten a woman’s health and increase the incidence of the woman’s illness and disability throughout the PPP. Understanding the link between PA and fatigue is particularly important for health care practitioners and decision makers who are involved in improving the current plans and developing effective therapeutic measures for promoting women’s health.

## Data Availability

The data is not publicly available because further research is being done and more manuscripts are being prepared. Data for the current study will be available upon reasonable request from the principal investigator or corresponding author.

## Abbreviations

PPP: Postpartum period
PPF: Postpartum fatigue
PPD: Postpartum depression
WHO: World Health Organization
PA: Physical activity
NCDs: Noncommunicable diseases
